# Stressing out the ER in aminoglycoside-induced hearing loss

**DOI:** 10.1038/cddis.2015.133

**Published:** 2015-05-14

**Authors:** P Garcia-Huerta, A Rivas, C Hetz

**Affiliations:** 1 Biomedical Neuroscience Institute, Faculty of Medicine, University of Chile, Santiago, Chile; 2 Program of Cellular and Molecular Biology, Center for Molecular Studies of the Cell, Institute of Biomedical Sciences, University of Chile, Santiago, Chile; 3 Department of Immunology and Infectious Diseases, Harvard School of Public Health, Boston, MA, USA

Aminoglycoside antibiotics are widely used in the treatment of a variety of bacterial infections.^1^ The use of aminoglycosides is usually limited to severe infections including those caused by multidrug-resistant tuberculosis. Nevertheless, in addition to their potent antimicrobial efficacy, all aminoglycosides can cause toxic side effects to kidney and inner ear, producing hearing loss, but they are still the most commonly prescribed antibiotics.^2^ Although damage inflicted by aminoglycosides to the kidney is usually reversible, alterations to the inner ear is permanent.^3^ Sensory hair cells in the inner ear are the primary receptors of auditory and vestibular information,^4^ and their degeneration causes hearing loss.^5^ Aminoglycoside antibiotics are well known to affect translational fidelity in bacteria and lower eukaryotes, altering the protein homeostasis (referred to as proteostasis),^3^ but only a few reports suggest that aminoglycoside antibiotics may also induce mRNA misreading in higher eukaryotes. However, despite the fact that protein-folding stress is a hallmark of the most common diseases affecting the nervous system,^6^ its contribution to ototoxicity remained unknown. Here Schatch *et al.*^7^ demonstrate, using genetic and pharmacological strategies, a functional contribution of the endoplasmic reticulum (ER) stress response to aminoglycoside-induced hearing loss.

The ER is the major subcellular compartment involved in protein folding and secretion. Many different perturbations in protein folding capacity, lead to a cellular state referred to as ER stress. ER stress engages an adaptive reaction to restore proteostasis known as the unfolded protein response (UPR). The UPR is initiated by the activation of three specialized stress sensors, where IRE1 is the most conserved among species (Figure 1a).^8^ Under conditions of chronic or irreversible ER stress, the UPR induces apoptosis through distinct overlapping signaling mechanisms, which include the upregulation of the transcription factor CHOP, the induction of oxidative stress, and the engagement of classical mitochondrial apoptosis pathway.^9^ Although aminoglycoside was shown to induce ER stress in kidney as a side effect^10^ due to chaperone inhibition,^11^ the mechanisms known to trigger hair cell apoptosis has mostly been associated with the inhibition of protein synthesis^5, 12^ and oxidative stress.^13^ Importantly, on an animal model of hearing loss driven by mitochondrial alterations, ER stress was suggested to have a key role.^14^

To define the cellular consequences of aminoglycoside treatment, Schacht *et al.* analyzed the changes in genome-wide gene expression patterns, uncovering a global effect on the ER proteostasis network. Then, to study the response of the auditory hair cells to ER stress, the authors used early postnatal mouse cochlear explants, observing that gentamicin increased the expression of CHOP in spiral ganglion cells (SGCs) but not in hair cell despite evident cytotoxicity (Figure 1b). To test the functional contribution of ER stress to ototoxicity, the authors took advantage of XBP1^+/−^ haploinsufficient mice. XBP1 is a master regulator of the UPR that control many genes involved in protein folding, secretion and quality control mechanisms. Remarkably, XBP1^+/−^ animals developed exacerbated loss of SGCs after gentamicin treatment. The decrease of these cells in the base of the cochlea correlated with the loss of synaptic connections to hair loss, providing an explanation for the observed auditory threshold deterioration after gentamicin treatment (Figure 1c). The authors went further and tested a pharmacological approach to mitigate ER stress in the context of aminoglycoside-mediated neurotoxicity. Chemical chaperones such as the bile acid tauroursodeoxycholic acid (TUDCA) have been widely employed to stabilize protein conformations and reduce ER stress levels of a variety of disease models.^15^ According to this, auditory physiology co-administration of TUDCA attenuated gentamicin-induced loss of auditory function. These results uncover a fundamental contribution of ER stress in the differential neuronal vulnerability of SGCs.

In contrast to the current study, in humans aminoglycoside ototoxicity has been linked to outer hair cell loss^13^ and small effects are described in SGCs. Importantly, it may be feasible that gentamicin induces ER stress in SGCs provoking synaptic dysfunction rather than cell death. On the other hand gentamicin may exert direct effects over hair cells without mediating UPR. Although this interesting study shed important lights on the role of XBP1, a precise definition of this aminoglycoside-induced ER stress may have enormous therapeutic potential and can be outlined by answering: (1) Is it just the induction of chaperones by XBP1s that alleviates ER stress under gentamicin treatment? (2) Is XBP1 the main UPR branch involved in maintaining the protein folding equilibrium in the presence of aminoglycoside-induced mistranslation? As many pharmacological^15^ and genetic tools^16^ are available to manipulate the UPR, additional studies are needed to define optimal therapeutic targets. Because UPR inhibitors are proposed as possible new strategies to kill cancer cells, it remains to be determined if hearing loss can be a secondary consequence of manipulating the UPR as a side effect. In summary, this study uncovers a high vulnerability of SGSs to ER proteostasis alterations as evidenced by the adverse secondary consequences of the alterations in translational fidelity triggered by aminoglycoside treatment.

## Figures and Tables

**Figure 1:**
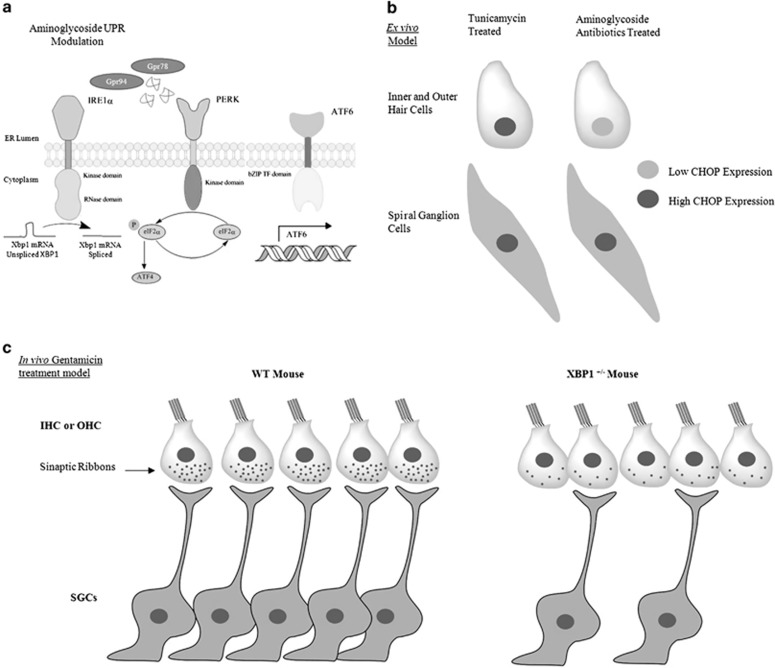
Models of aminoglycoside-induced ototoxicity. (**a**) Aminoglycosides activate the UPR system in cell culture. (**b**) Cochlear explant treated with geneticin undergoes ER stress. (**c**) XBP1 haploinsufficiency (XBP1^+/−^) specifically affects SGCs density and synaptic connections (synaptic ribbons)
